# Endoscopic Full-Thickness Resection of Rectal Gastrointestinal Stromal Tumor With Endoscopic Rectal Reconstruction

**DOI:** 10.14309/crj.0000000000002174

**Published:** 2026-06-02

**Authors:** Azhar Hussain, Abinash Subedi, Fathima K. Suhail, Bishnu Sapkota, Hafiz Muzaffar A. Khan

**Affiliations:** 1 Division of Medicine, State University of New York Upstate Medical University, Syracuse, NY; 2 Division of Gastroenterology and Hepatology, State University of New York Upstate Medical University, Syracuse, NY; 3 Center for Interventional Endoscopy (CIE), AdventHealth, Tampa, FL

**Keywords:** rectal gastrointestinal stromal tumor, endoscopic full-thickness resection, endoscopic full-thickness resection

## Abstract

Gastrointestinal stromal tumors (GISTs) of the rectum are rare and present unique management challenges due to their proximity to vital pelvic organs. This report describes the case of a 73-year-old man with multiple comorbidities who was diagnosed with a 3.1 cm distal rectal gastrointestinal stromal tumor. Successful exposed endoscopic full-thickness resection was performed resulting in complete excision of the gastrointestinal stromal tumor and complete endoscopic rectal reconstruction was performed using endoscopic overstitch device. This case demonstrates the effectiveness of free hand endoscopic full-thickness resection as a minimally invasive treatment of rectal GISTs and other subepithelial lesions, particularly in patients for whom traditional surgery poses elevated risks.

## INTRODUCTION

Gastrointestinal stromal tumors (GISTs) are uncommon mesenchymal tumors of the gastrointestinal tract, with rectal GISTs accounting for about 5% of cases.^1^ Their location presents unique challenges, as tumors in the anterior rectum are often close to vital pelvic structures, potentially affecting anorectal function and complicating surgical management. For most localized GISTs, treatment options include surgical resection and pharmacological therapy. Tyrosine kinase inhibitors (TKIs) are commonly used to shrink tumors preoperatively or reduce recurrence risk after resection.^2^ Surgical resection remains the primary intervention for resectable tumors,^3^ while advancements in endoscopic techniques have introduced endoscopic full-thickness resection (EFTR) as a minimally invasive alternative. EFTR enables removal of tumors originating from deeper layers while minimizing morbidity and preserving anorectal function through real-time defect closure.^4^ In addition, low rectal lesions provide a unique opportunity for safe endoscopic resection given its relationship to the peritoneal reflection.

This report presents a case of a rectal gastrointestinal stromal tumor (GIST) successfully managed using freehand EFTR technique, demonstrating its feasibility in patients with significant comorbidities where traditional surgical intervention poses higher risks. The minimally invasive nature of EFTR makes it an ideal option for patients who may not tolerate open or laparoscopic surgery. In this case, the patient's comorbidities and the tumor's location warranted a tailored approach, allowing EFTR to effectively balance oncological control with reduced recovery time and minimal procedural complications.

## CASE REPORT

**Figure 1.**
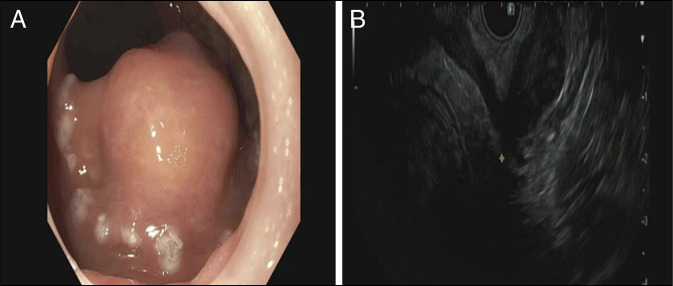
Endoscopic (A) and endoscopic ultrasound (B) view of the 3.1 × 2.1 cm mass along the anterior wall of the distal rectum arising from the muscularis propria.

A 73-year-old man with a past medical history of stage 3b chronic kidney disease, hypertension, hyperlipidemia, type 2 diabetes mellitus, and benign prostatic hyperplasia was evaluated for an increasing prostate-specific antigen value. Magnetic resonance imaging revealed no focal prostate lesions but incidentally identified a 3.1 × 2.1 cm mass along the anterior wall of the distal rectum. The patient subsequently underwent a colonoscopy and lower endoscopic ultrasound with fine needle biopsy (Figure 1). Fine needle biopsy of the rectal mass confirmed a diagnosis of GIST. Patient underwent positron emission tomography computed tomography from skull to thigh which showed 3.1 × 2.1 cm (AP × transverse view) metabolically active soft tissue lesion involving the mid anterior rectal wall, consistent with known rectal GIST. No evidence of fluorodeoxyglucose avid metastatic lesions/lymph nodes. Patient was referred to oncology team, but he decided against any neoadjuvant therapy. A multidisciplinary discussion involving colorectal surgery, urology, oncology and gastroenterology determined that freehand EFTR would be the preferred approach, given patient's comorbidities, tumor location and patient's preferences to avoid surgery.

On the day of the procedure, laboratory analysis revealed normocytic anemia with hemoglobin 12.0 g/dL elevated creatinine of 2.24 mg/dL, and an estimated glomerular filtration rate of 30 mL/min/1.73 m^2^. A freehand EFTR was performed, achieving complete removal of the rectal GIST. Endoscopic rectal reconstruction was performed using the OverStitch suturing device, with no immediate complications.

### Procedure details

**Figure 2.**
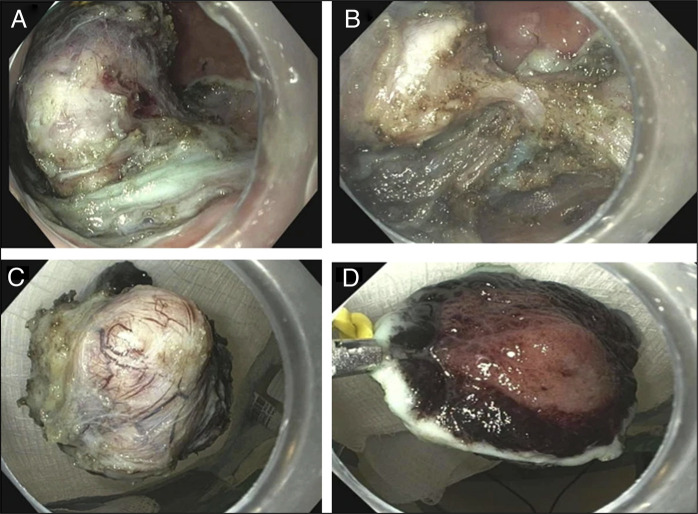
Endoscopic view of dissection of the lesion from the underlying muscularis propria with an electrocautery knife (A) and visualization of lesion attachment to muscularis propria with thick, and broad-based stalk (B). Complete retrieval of the dissected lesion with visible full-thickness defect of 3.3 cm before closure (C) and (D).

The procedure was performed under general anesthesia. A forward viewing gastroscope with a clear cap was advanced to the distal rectum, where lesion was identified. Hetastarch with methylene blue was used to create a submucosal cushion around the lesion. A circumferential incision into the submucosa around the lesion was made using an ERBE Hybrid I type knife and an insulated tip knife using standard endoscopic submucosal dissection technique using electrosurgical settings of Endocut Q, Effect 2, forced coagulation 2/50. The lesion was then carefully dissected from the underlying deep layers using the same knives (Figure 2). It was attached to the muscularis propria by a thick, broad-based stalk, which was subsequently dissected, ensuring preservation of the capsule (Figure 2). This resulted in a medium-sized full-thickness defect (50 × 30 mm area), and the specimen was retrieved using a Roth net (Figure 2). Hemostasis was achieved using electrocautery knife, and CO_2_ insufflation used during the procedure.

**Figure 3.**
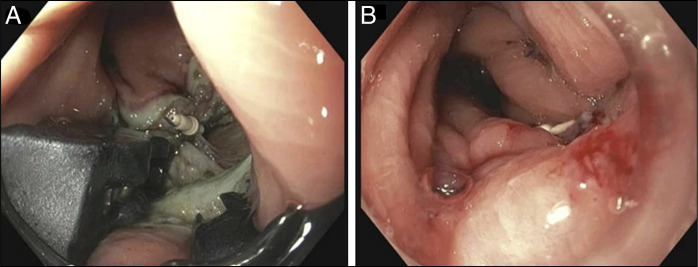
(A) and (B) Endoscopic closure being performed using OverStitch suturing device and defect was closed in multiple layers using 2-0 polypropylene suture.

Given the full-thickness nature of the resection, endoscopic closure was performed using OverStitch suturing device. The defect was closed using a 2-0 polypropylene suture in a running fashion, first approximating the muscle layer, followed by the mucosal layer to achieve complete rectal reconstruction (Figure 3). A total of 6 sutures were placed with cinches at both ends. Excellent tissue approximation was achieved, and no bleeding was observed upon completion of the procedure. Given large defect size and full-thickness entry coupled with difficult location, good endoscopic closure with optimal suture tension and endoscopic reconstruction of neorectum to prevent luminal narrowing was needed, so we used running multilayer OverStitch technique over interrupted sutures or clip-assisted closure. Patient received 1 dose of piperacillin/tazobactam for surgical prophylaxis. Total procedure time was 4.5 hours.

### Patient outcomes

The patient tolerated the procedure well and was admitted for overnight observation. He remained hemodynamically stable and started on a clear liquid diet for 2 days before advancing to a regular diet. He was discharged the day after the procedure with plans for post-resection surveillance, including a follow-up colonoscopy for 13 months.

**Figure 4.**
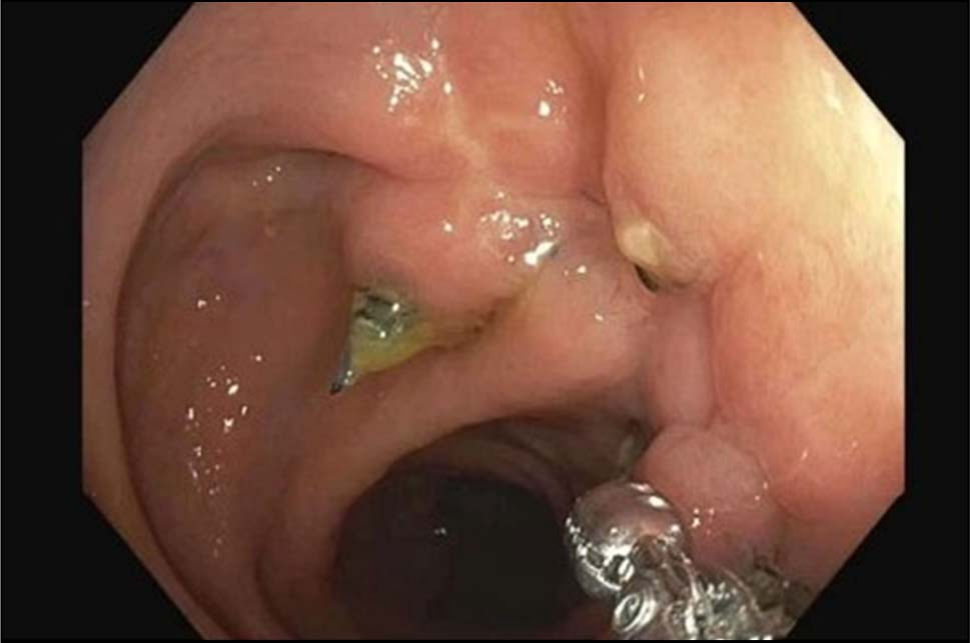
Surveillance endoscopic ultrasound at 6 months follow-up revealing well-healed resection site with healthy-appearing mucosa.

Surgical pathology confirmed unifocal spindle cell type GIST with pT2 per pTNM classification and a histologic grade of G1 (low grade per formal risk stratification) with negative margins, and mitotic rate of 1 mitosis per 5 mm^2^. After 6 months of follow-up, surveillance endoscopic ultrasound revealed a well-healed resection site with cinches and suture at the anastomosis with healthy-appearing mucosa (Figure 4). There was no evidence of residual or recurrent disease endoscopically or on repeat biopsy of the resection site. At 13 months of follow-up, he underwent magnetic resonance imaging for surveillance postendoscopic resection and did not reveal disease recurrence. Oncology recommended against adjuvant therapy and recommended conservative management and surveillance per National Comprehensive Cancer Network guidelines.

## DISCUSSION

Management of GISTs depends on prognostic factors such as tumor size, location, mitotic index, and risk of malignancy.^5^ In certain cases, pharmacological therapy, particularly tyrosine kinase inhibitors (TKIs), may complement surgical or endoscopic approaches. Surgical resection, including transanal minimally invasive surgery and laparoscopic approaches, has been the traditional treatment modality. EFTR is emerging as a viable, novel, and minimally invasive alternative for selecting patients with subepithelial lesions as per the American Gastroenterological Association 2024 practice updates.^6^ There are several EFTR techniques including freehand EFTR technique (for larger lesions > 3 cm) and nonexposed techniques including submucosal tunneling endoscopic resection and full-thickness resection device (for smaller lesions ≤ 3 cm). Compared with surgical resection via minimally invasive surgery and laparoscopic approaches, EFTR offers several advantages over conventional surgery, including reducing procedural morbidity, shorter recovery times, and preservation of anorectal function. This is a safe and effective technique that allows for complete resection of lesions.^7^ The multidisciplinary decision to use freehand EFTR technique in this case was guided by the patient's significant comorbidities, the tumor size, low rectal location, and patient's preference to avoid major surgical intervention.

Our case report is unique and novel because of the complexity of the patient comorbidities, tumor size (>3 cm), the distal rectal location, use of the freehand exposed technique in difficult rectal location, and the multilayer endoscopic reconstruction with good postprocedure patient outcomes. This case highlights the growing role of EFTR with freehand technique in managing rectal GISTs, particularly in patients who are considered at high risk of surgery. Finally, while we have demonstrated short-term endoscopic healing and absence of early recurrence, rectal GISTs carry a risk of delayed local recurrence or distant metastasis that may occur years after resection, needing future surveillance. Further studies are needed to compare long-term outcomes of EFTR vs surgical approaches in rectal GIST resection.

## DISCLOSURES

Author contributions: All authors have substantial contributions to conception/design, drafting, or critically revising the work for intellectual content; final approval of the version to be published; and agreeing to be accountable for all aspects of the work. A. Hussain is the article guarantor.

Financial disclosure: None to report.

Informed consent was obtained for this case report.

## ABBREVIATIONS:

EFTR: endoscopic full-thickness resection
GIST: gastrointestinal stromal tumors
SELs: subepithelial lesions
TKIs: tyrosine kinase inhibitors
